# Safety and efficacy of lentinan nasal drops in patients infected with the variant of COVID-19: a randomized, placebo-controlled trial

**DOI:** 10.3389/fphar.2023.1292479

**Published:** 2023-12-01

**Authors:** Wenhan Fan, Benming You, Xinyu Wang, Xu Zheng, Aijing Xu, Yangang Liu, Haoran Peng, Wei Yin, Mingxiao Xu, Xu Dong, Yayun Liu, Ping Zhao, Xuesong Liang

**Affiliations:** ^1^ Department of Infection Diseases, First Affiliated Hospital of Navy Military Medical University, Shanghai, China; ^2^ Department of Pharmacy, First Affiliated Hospital of Navy Military Medical University, Shanghai, China; ^3^ Department of Microbiology, PLA Key Laboratory of Biodetection and Biodefense, Shanghai Key Laboratory of Medical Biodefense, Navy Military Medical University, Shanghai, China

**Keywords:** lentinan, COVID-19, cohort study, mucosal immunity, nasal drops

## Abstract

**Objective:** Lentinan has antiviral, anti-tumor, immunomodulatory, stimulating interferon production, and other pharmacological effects. Previous animal experiments have shown that lentinan nasal drops can assist [Corona Virus Disease 2019) COVID-19] vaccine to induce high levels of neutralizing antibodies and can effectively resist the invasion of severe acute respiratory syndrome coronavirus 2 (SARS-CoV-2). This study aimed to evaluate the safety and efficacy of lentinan nasal drops in patients infected with Omicron (SARS-CoV-2 variant) through a dose-escalation study and a placebo-controlled trial.

**Methods:** A randomized, placebo-controlled trial. The study was divided into two phases: Phase I: a dose escalation trial in which 24 COVID-19 patients were enrolled, that is, 12 in the escalation dose group (50, 75, and 100 µg/day) and 12 in the standard treatment group. The aim was to evaluate the safety and tolerance of lentinan nasal drops. The second stage was a placebo-controlled study. The optimal dose group of the first stage was used as the therapeutic dose, and the sample size was expanded to verify the anti-COVID-19 efficacy of lentinan nasal drops.

**Results:** In the dose-increasing study, lentinan nasal drops showed good safety, and no serious adverse reactions occurred. The virus shedding time of the 100 µg dose group was significantly shorter than that in the control group (7.75 ± 1.71 VS 13.41 ± 3.8 days) (*p* = 0.01), and the 100 µg/day lentinan nasal drops were tolerated well. The results of the placebo-controlled study showed that compared with that in the placebo group, the time for COVID-19 antigen to turn negative was significantly shorter in the 100 µg lentinan nasal drop group (*p* = 0.0298), but no significant difference was observed in symptom improvement between the two groups. In the placebo-controlled study, two patients experienced mild nasal discomfort with nasal drops, but the symptoms relieved themselves.

**Conclusion:** Lentinan nasal drops are tolerated well and can shorten the time of virus clearance.

## 1 Introduction

COVID-19 caused by SARS-CoV-2 has spread widely around the world, which resulted in a profound disaster to human society (https://www.gov.cn/zhengce/zhengceku/2022-03/15/content_5679257.htm). Although the World Health Organization declared on 5 May 2023 that the COVID-19 pandemic is no longer a “public health emergency of international concern,” it does not mean that the COVID-19 pandemic is over. The global risk of COVID-19 remains high, and the threat to human health still exists. We still need to work hard to find drugs for the treatment and control of COVID-19.

At present, two main therapeutic strategies have been used for COVID-19. One therapeutic strategy is to use small-molecule antiviral drugs, such as paxlovid, remdesivir, and molnupiravir. Although numerous small-molecule drugs are largely effective in preventing severe infection of coronavirus, several of them also have a high incidence of drug interaction, drug-induced liver function injury, and other adverse events (AEs) during use (Wang et al., 2020; Wang et al., 2022). Another type of drugs represented by monoclonal antibodies, including bamlanivimab, bamlanivimab/etesevimab, casirivimab/imdevimab, *etc.*, also showed greatly decreased neutralizing activity due to the high variability of SARS-CoV-2 (Cao et al., 2022a; Scotto et al., 2022; Tao et al., 2022).

On the other hand, the frst site invaded by SARS-CoV-2 is the upper respiratory tract. Nasal vaccines and drugs have been already in diferent stages of development. Apart from prophylactic purposes, mucosal immunity can be exploited for therapeutic purposes too (Tandon et al., 2022; Chavda et al., 2023).

The main component of lentinan is β-glucan, which has multiple clinical pharmacological effects, such as antiviral, anti-tumor, immunomodulatory, and stimulating interferon production (Haba et al., 1976; Deng et al., 2018; Ren et al., 2018; Murphy et al., 2020; Zi et al., 2020). In addition, β-glucan can be used as a potential vaccine adjuvant or immune training agent to prevent or treat COVID-19 (Córdova-Martínez et al., 2021). A significant reduction in clinical symptoms was observed after the administration of nasal drops of resveratrol plus carboxymethyl-β-glucan in infants under 6 months of age who had a common cold caused by rhinoviruses (Baldassarre et al., 2020). However, few clinical cohort studies have focused on the use of lentinan in COVID-19 patients.

Our preliminary experiment using SARS Cov-2 envelope spike protein mixed with lentinan solution administered as nasal drops in Syrian golden hamsters effectively induced the production of anti-coronavirus spike protein receptor-binding domain immunoglobulin G (IgG) in the hamsters and effectively protect them against coronavirus infection (International Application No: PCT/CN202/101512) (Supplementary Data S2). Based on the above findings, we plan to explore the safety and efficacy of lentinan nasal drops against COVID-19 through dose escalation and placebo-controlled studies.

## 2 Methods

This study was divided into two phases. The first phase was the dose-climbing trial whose main purpose was to test the safety and adverse effects of the drug and explore the maximum effective amount of nasal drops. The second phase was based on the results of the dose-climbing study and further conducted a randomized placebo-controlled study to investigate the antiviral efficacy and clinical symptom relief of lentinan nasal drops. This study was approved by the Ethics Committee of Shanghai Changhai Hospital (CHEC2022-111) and registered in the Chinese Clinical Trials Registry (No: ChiCTR2300070776). The study was carried out in accordance with the 1964 Declaration of Helsinki and its later amendments.

### 2.1 Trial drug

**FIGURE 1:**
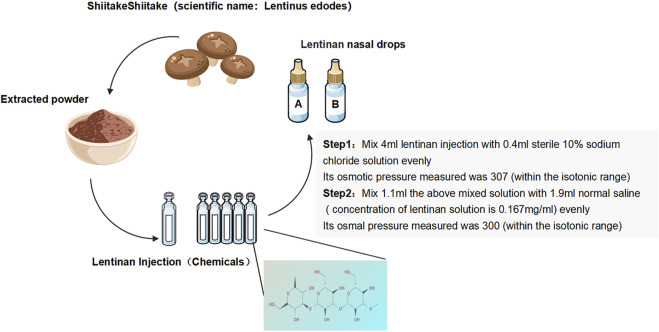
Preparation of lentinan nasal drops.

Lentinan nasal drops: Lentinan nasal drops (Jinling Pharmaceutical) (National Drug license No. H20030131) were adjusted in accordance with the production standards or specifications of human nasal drops. Their marketing license was from the National Engineering Technology Research Center for Emergency Prevention and Control Drugs (specification: 2 mL:1 mg). In reference to the literature and combined with the recommendations of pharmacological experts (Wang et al., 2020), the final osmotic pressure of lentinan nasal drops was 300 mmol/L, and the final concentration was 0.167 mg/mL (Figure 1).

Placebo: An equal dose of saline was used for the placebo.

### 2.2 Research design

#### 2.2.1 Phase 1 (dose escalation)

The inclusion criteria were as follows: 1) 18–60 years old, 2) gender not limited; 3) SARS-CoV-2 infection was confirmed by detecting SARS-CoV-2 nucleic acid in nasopharyngeal swab samples by (real time-polymerase chain reaction, RT-PCR) with or without COVID-19-related symptoms within 5 days before enrollment (The virus isolation was sequenced and submitted to GenBank [ON965380; ON965371; ON965362; ON965361] (Supplementary Data S1).

The exclusion criteria were as follows: 1) COVID-19 reinfection, 2) bleeding tendency (PLT≤90 × 109/L or INR≥1.5×ULN) 3) allergy to lentinan and its pharmaceutical ingredients, 4) severe allergic reactions, including systemic urticaria, angioedema, and allergy; 5) nasal mucosal lesions, 6) received plasma therapy and SARS-CoV-2 antibody therapy while recovering from COVID-19, 7) received other investigational drugs for the treatment of SARS-CoV-2, 8) pregnant or lactating women, 9) confirmed liver cirrhosis through CT, B-ultrasound, or MRI examination; alanine aminotransferase or aspartate aminotransferase more than five times the upper limit of normal; known severe renal impairment (estimated glomerular filtration rate <30 mL/min per 1·73 m^2^) or receipt of continuous renal replacement therapy, haemodialysis, or peritoneal dialysis; history of structural lung disease, obstructive pulmonary disease, asthma; Cardiovascular disease such as ischemic heart disease, history of tachycardia or bradycardia requiring treatment, adams-stokes syndrome, *etc.* 10) assessment by researchers who were unsuitable to participate in this study.

##### 2.2.1.1 Research group

The protocols for the lentinan group were as follows: 1) 50 µg group: 50 µg/day, 1 drop per nasal cavity (about 100 μL, 0.167 mg/mL),Q8h, and total course of treatment of 5 days; 2) 75 µg group: 75 µg/day, 1 drop per nasal cavity (about 100 μL, 0.167 mg/mL), Q5h, and total course of treatment of 5 days; 3) 100 µg group: 100 µg/day, 1 drop per nasal cavity (about 100 μL, 0.167 mg/mL), Q4h, and total course of treatment of 5 days. Routine treatment was performed in accordance with the diagnosis and treatment protocol for coronavirus pneumonia. All admitted patients received only standard care (SC) recommended by the Chinese COVID-19 prevention and treatment program, including nonsteroidal antiinflammatory drugs, paracetamol (if body temperature ≥ 38°C), and cough mixture (licorice mixture, a traditional Chinese medicine) (trial version 9) (https://www.gov.cn/zhengce/zhengceku/2022-03/15/content_5679257.htm).

The control group was given the same dose of placebo drops and received conventional treatment following the diagnosis and treatment protocol for coronavirus pneumonia (trial 9 version).

#### 2.2.2 Phase 2 (sample expansion)

**FIGURE 2:**
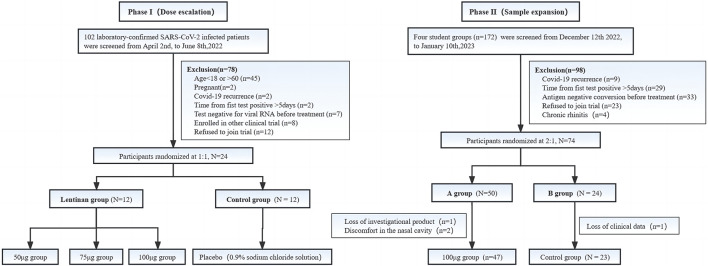
The screening process for participants in two phases.

The optimal dose (100 µg/day) was selected based on the results of the first-phase dose-escalation test. The sample size was expanded to conduct a 2:1 randomized placebo-controlled study. (Figure 2).

#### 2.2.3 Procedures

##### 2.2.3.1 Randomization and blinding

A table of random numbers was generated using SPSS (version 25.0). Patients were randomized into the experimental and control groups at the ratio of 2:1. In the phase II study, a clinical symptom registry was developed and distributed to enrolled patients to assess the remission of clinical symptoms in the two groups. The enrolled patients were instructed to perform standard nasal drops and trained to fill out this registry according to their own antigen test results. Opaque envelopes that contained either A or B strips of paper were distributed to the patients in the order generated by the random table of numbers. During the study, the results of antigen detection for COVID-19 were recorded on days 0, 1, 3, 5, and 7. Registries were collected at the end of the study.

##### 2.2.3.2 Endpoints

Endpoints of Phase I: The primary endpoints were the evaluation of the safety of medication, patient tolerability during medication, and the incidence of adverse events (AEs) and serious AEs (SAEs) after medication. The secondary endpoint was the time the virus turned negative. After the patients were enrolled, nasopharyngeal swabs were collected every day, and SARS-CoV-2 virus was quantitatively detected by real time-polymerase chain reaction (RT-PCR) until the nucleic acid results turned negative. Negative nucleic acid results were defined as two consecutive negative results (ORF1ab and N gene Ct values >35). The time of virus negative conversion was defined as the time of the first negative nucleic acid after two consecutive negative nucleic acids.

**FIGURE 3:**
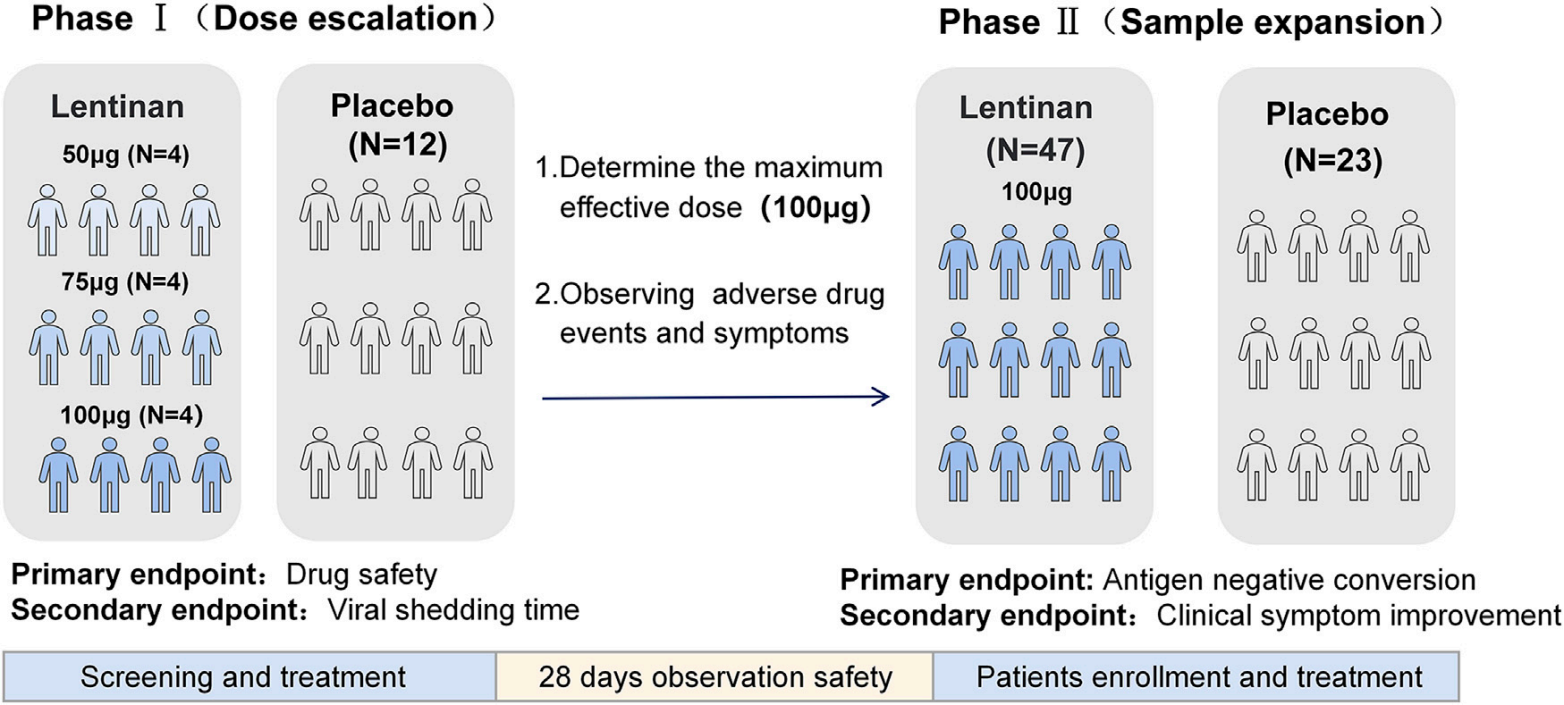
Flowcharts of two phases of the experiment.

Endpoints of Phase II: The primary end point was the observation and evaluation of the negative conversion rate tested by antigen on day 5(Supplementary Figure S1). Antigen negative conversion was defined as two consecutive negative results. The secondary endpoint was the observation of the remission of clinical symptoms in both groups (Note: Safety data of both phases were only collected after the enrolled patients have been treated) (Figure 3).

### 2.3 Statistical analysis

All statistical analyses were conducted using R 4.4.2 from Windows (R Foundation for Statistical Computing, Vienna, Austria) (https://www.r-project.org). The Kolmogorov–Smirnov test was used to evaluate whether the sample data were distributed normally. For continuous variables, data were presented as mean ± standard deviation or median with an interquartile range, and they were analyzed by Student’s t-test or Mann–Whitney *U* test of the different groups. For categorical variables, data were presented as frequencies or percentages and compared between groups as appropriate by Pearson chi-square or Fisher’s exact test. A two-sided *p* < 0.05 was considered statistically significant.

## 3 Result

### 3.1 Phase 1

In this phase, 102 patients admitted to our hospital due to COVID-19 were initially screened, and 24 patients were included in this phase of the study. These 24 patients were randomly enrolled at 1:1 ratio, that is, 12 patients in the lentinan group and 12 in the clinical control group. Baseline indicators were well comparable between the two groups (Table 1).

**TABLE 1:** Comparison of baseline characteristics and nucleic acid clearance time between lentinan subgroups and control group.

Variables	LNT(n = 12)	control(n = 12)	t/χ2	*p*-value
**Characteristics**				
Male, n (%)	2 (16.67)	1 (8.33)	—	1
Age, Mean ± SD, years	37.25 ± 10.45	38.16 ± 10.65	−0.21	0.833
BMI	20.74 ± 2.26	22.61 ± 2.63	−1.87	0.075
**Vaccination status against COVID-19, n (%)**				
Partially vaccinated	4 (33.33)	6 (50)	0.171	0.679
Full vaccination	8 (66.66)	6 (50)		
**Clinical indicators**				
White blood cell (9×10^9^/L)	4.54 ± 1.18	4.57 ± 1.19	−0.079	0.937
Lymphocyte count (9×10^9^/L)	1.43 ± 0.46	1.66 ± 0.99	−0.713	0.483
Neutrocyte (9×10^9^/L)	2.56 ± 1.06	2.38 ± 1.45	0.355	0.726
CRP (mg/L)				
≥10	4 (33.33)	2 (16.67)	0.222	0.637
<10	8 (33.33)	10 (83.33)		
**Initial SARS-CoV-2 RNA level (CT value)**				
ORF1ab, mean ± SD	22.5 ± 4.32	19 ± 5.09	1.81	0.083
N, mean ± SD	20.42 ± 6.68	16.83 ± 4.8	1.508	0.145
Nucleic acid shedding time	9.67 ± 2.64	13.41 ± 3.8	−2.807	0.01

Abbreviation: SD, standard deviation; BMI: Body Mass Index; CRP:C-reactive protein; CT, value:Viral RNA, test cycle threshold; LNT:lentinan group; Full vaccination were defined as those with at least 2 doses of Comirnaty or 3 doses of CoronaVac.

All enrolled patients showed clinically mild conditions without severe symptoms. During the study period, all 24 patients received symptomatic treatment, including non-steroidal anti-inflammatory drugs and cough suppressants, and none received antiviral drugs, glucocorticoids, or monoclonal antibodies other than the study drugs. The 12 patients enrolled in the lentinan group were randomly treated with different doses of experimental drugs for 5 days (50, 75, and 100 µg/day).

Lentinan nasal drops were well tolerated, and the incidence of secondary or higher side effects was 0. After 28 days of follow-up, all patients in the lentinan group did not show symptoms of nasal discomfort, rhinitis, nor decreased taste and smell. Meanwhile, the median time of virus clearance in the lentinan group was significantly shortened (9.67 ± 2.64 and 13.42 ± 3.8 days, *p* = 0.01) (Table 1).

**FIGURE 4:**
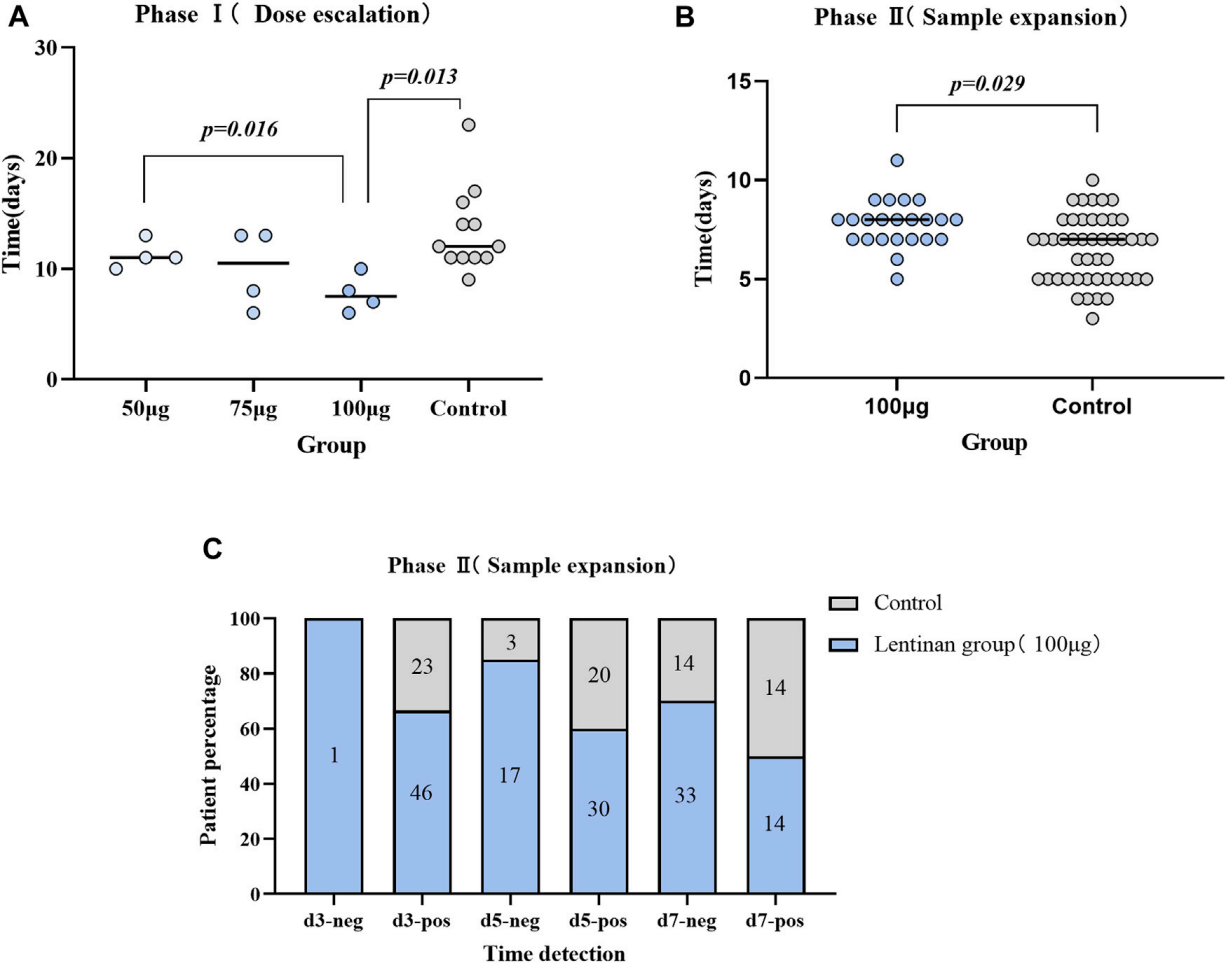
Comparison of antiviral efficacy between different groups. **(A)** Comparison of nucleic acid negative conversion time between subgroups of Lentinan group and control group (Phase I trial); **(B)** Comparison of Antigen negative conversion time between lentinan 100 μg group and placebo group (Phase II trial); **(C)** Comparison of time node of antigen negative conversion rate between lentinan 100 μg group and placebo group on day 3,5 and 7.

Comparisons between each two of the three dose subgroups and the control group showed that the anti-COVID-19 efficacy of lentinan was significantly dose dependent, but only the 100 µg dose group had a significant difference compared with the control group (7.75 ± 1.71 vs. 13.42 ± 3.8 days, *p* = 0.013) (Table 2) (Figure 4A).

**TABLE 2:** Comparison of the viral shedding time between each lentinan subgroup and the control group (Phase 1).

LNT subgroup	Time (days)	Control	t	*p*-value
Group A	11.25 ± 1.26	13.41 ± 3.8	−1.089	*p* = 0.291
Group B	10 ± 3.56		−1.578	*p* = 0.137
Group C	7.75 ± 1.71		−2.836	*p* = 0.013
Group A vs. Group B			0.662	*p* = 0.532
Group A vs. Group B			3.3	*p* = 0.016
Group B vs. Group C			1.14	*p* = 0.289

LNT, lentinan group; Group A: 50 μg/day,Q8h, one drop in each side of the nasal cavity (about 100 μL, 0.167 mg/mL), total course of treatment is 5 days; Group B: One drop in each nasal cavity (about 100μL, 0.167 mg/mL), Q5h, total course of treatment is 5 days; Group C: One drop in each lateral nasal cavity (about 100 μL, 0.167 mg/mL), Q4h, total course of treatment is 5 days.

### 3.2 Phase 2

According to the results of the first phase of the study, the optimal dose of lentinan in the second phase was 100 µg/day. A total of 172 COVID-19 patients from four classes of two universities in Shanghai participated in screening, and 74 patients of COVID-19 primary infection were randomly assigned to the lentinan treatment and placebo control groups at 2:1 ratio. Of these 74 patients, 2 were excluded because of missing data, and 2 voluntarily withdrew from the study due to nasal discomfort (which was relieved spontaneously in a short time). Finally, the data of 70 patients, including 47 patients in the lentinan group and 23 patients in the placebo group, were included.

All the enrolled patients had clinically mild conditions without severe symptoms. During the study period, all 70 patients received symptomatic treatment, including non-steroidal anti-inflammatory drugs and cough suppressants, and none received antiviral drugs, glucocorticoids, or monoclonal antibodies other than the study drugs.

No significant differences were observed in the gender, age, vaccination, and other baseline characteristics between lentinan group and placebo groups. We further observed a statistically significant difference between the antigen negative conversion rate of 100 µg lentinan group and the control group on days 5 and 7 (*p* = 0.044, *p* = 0.013; Table 3; Figure 4C). Moreover, the median of antigen negative conversion time for the lentinan and placebo groups were 7 (confidence interval (CI)%: 5, 8) and 8 days (CI%: 6.5, 8), respectively, and their difference was statistically significant (*p* = 0.029) (Figure 4B). In addition, we followed up the clinical symptoms of the patients on days 1, 5, and 7 after medication, including fever, runny nose, cough, diarrhea, and changes in smell and taste, and found no statistically significant differences between the two groups (Table 4) (Supplementary Figure S2).

**TABLE 3:** Comparison of baseline characteristics and viral shedding rate at each time point between lentinan group and placebo group tested by antigen (Phase 2).

Variables	LNT (N = 47)	Pbo(N = 23)	t/χ2	*p*-value
Characteristics				
Vaccination,n (%)			0.225	0.635
Partially vaccinated	17 (36.2)	7 (30.4)		
Full vaccination	30 (63.8)	16 (69.6)		
Age,Mean ± SD,years	20.68 ± 1.67	20.56 ± 1.34	0.289	0.773
Male,n (%)	43 (91.49)	19 (82.61)	0.486	0.485
Antigen dectection,n (%)				
d3-neg	1 (2.1)	0 (0)		
d5-neg	17 (36.2)	3 (13)	4.047	0.044
d7-neg	33 (70.2)	14 (60.9)	6.217	0.013

LNT, lentinan group; Pbo, placebo group; d3-neg, Antigen conversion rate on the third day.

**TABLE 4:** Comparison of common clinical symptoms between the lentinan group and the placebo group at each time point.

	day1			day5			day7		
Symptoms,n (%)	LNT (N = 47)	Pbo (N = 23)	*p*-value	LNT (N = 47)	Pbo (N = 23)	*p*-value	LNT (N = 47)	Pbo (N = 23)	*p*-value
Fever	35 (74.5)	19 (82.6)	0.446	6 (14)	2 (8.7)	0.918	3 (6.4)	1 (4.3)	1
runny nose	44 (93.9)	18 (78.3)	0.134	36 (83.7)	17 (73.9)	0.806	24 (51.1)	13 (56.5)	0.667
Cough/Sore throat	45 (95.7)	18 (78.3)	0.062	39 (90.7)	19 (82.6)	1	17 (36.2)	9 (39.1)	0.809
Nausea/Vomiting	20 (42.6)	6 (26.1)	0.181	5 (11.6)	2 (8.7)	1	3 (6.4)	2 (8.7)	1
Diarrhea	13 (27.7)	8 (34.8)	0.541	5 (11.6)	3 (13)	1	4 (8.5)	3 (13)	0.865
Loss of smell	16 (34)	6 (26.1)	0.5	10 (23.3)	4 (17.4)	0.95	10 (21.3)	3 (13)	0.405
Loss of taste	14 (28.9)	7 (30.4)	0.956	11 (25.6)	5 (21.7)	0.702	10 (21.3)	4 (17.4)	0.949
Muscular soreness	36 (76.6)	16 (69.6)	0.527	12 (27.9)	3 (13)	0.376	5 (10.6)	2 (8.7)	1
Headache	40 (85.1)	17 (73.9)	0.26	10 (23.3)	3 (13)	0.612	6 (12.8)	2 (8.7)	0.918
Fatigue	35 (74.5)	18 (78.3)	0.728	20 (46.5)	2 (8.7)	0.01	13 (27.7)	2 (8.7)	0.069
Shortness of breath	15 (31.9)	4 (17.4)	0.199	9 (20.9)	3 (13)	0.765	4 (8.5)	2 (8.7)	1

LNT, lentinan group; Pbo, placebo group.

## 4 Discussion

This study aimed to determine the safety, tolerability, and the effective dose of nasal drops through a two-stage clinical trial. In addition, we intended to further evaluate the antiviral efficacy of nasal drops in COVID-19 patients. Through a large number of literature searches, we discovered a few reports on the treatment of COVID-19 by intravenous injection but no study on the treatment by nasal drops. Therefore, this study is the world’s first clinical research on the use of lentinan nasal drops in the treatment of COVID-19, which is both innovative and practical.

In our two-phase study, the lentinan nasal drops were shown to be safe and effective against coronavirus. Our preliminary experimental results showed that high levels of IgG antibodies could be induced in the blood of Syrian golden hamsters by nasal drops consisting of SARS-CoV-2 envelope spike protein and lentinan. We speculate that the lentinan exerts its antiviral activity by activating the mucosal immune response in the nasal cavity, although the exact mechanism remains to be experimentally determined.

The nasopharynx is the first target site for coronavirus to enter the human body, which means that the nasal antibodies produced by mucosal immunization play an important role in effectively neutralizing upper respiratory viruses. An increasing number of studies have focused on the role of mucosal immunity in the treatment and prevention of respiratory infection. A recent study collected plasma and nasal samples from 446 adults hospitalized for COVID-19 1 year after discharge and tracked how long their nasal and plasma antibody levels persisted. Studies have shown that at 4 weeks after infection, antibodies in the nasal cavity appeared, and after 3–9 months, the level of antibodies in the nasal mucosa gradually decreased, but the resistance to the mutant strain Omicron was shorter. The researchers believe that vaccination is effective in producing and boosting antibodies in the blood but has negligible effect on nasal IgA levels, suggesting that intramuscular vaccination is unlikely to evoke a nasal mucosal response. Infection prevention and transmission require a substantial boost in nasal antibodies. Intranasal and aerosolized vaccines have shown the greatest promise in this regard (Liew et al., 2023). An animal study showed that hamsters produced high titers of coronavirus-specific antibodies after nasal inoculation with a coronavirus spike protein vaccine expressed by a human parainfluenza virus type 3 vector. The hamsters’ lungs were induced to produce a robust memory T cell response, which formed an infection protective barrier in the lungs and further confirmed the effectiveness of nasal mucosal immunity (Ilinykh et al., 2022). This result is also consistent with that of our previous hamster experiment with lentinan. Another study has shown that the mucosal immune memory was induced by the injection of adjuvant-free spike proteins or mRNA into the nasal cavity of hamsters, which allowed CD8+T cells, CD4^+^ T cells, and B cells to remain in the tissue and strongly induced the production of mucosal IgA and lgG; thus, the hamsters received a certain immune preservation effect (Mao et al., 2022). Other studies have shown that the autonomic immunity of mucosal epithelial cells is a powerful barrier against viral infection. Through immune training, this innate immune defense ability can be enhanced (van der Meer et al., 2015; Ross and Herzberg, 2016; Divangahi et al., 2021; Ziogas and Netea, 2022). Therefore, searching for a mechanism that enhances resistance and reduces virus transmission by enhancing mucosal immunity is important.

At present, most of the drugs used to treat coronavirus are administered orally or intravenously, and most vaccination methods are intramuscular injection. It can’t be denied that drugs, such as paxlovid, remdesivir, and molnupiravir, etc., and drugs represented by monoclonal antibodies, including bamlanivimab, bamlanivimab/etesevimab, casirivimab/imdevimab, etc., play a positive role in the field of antiviral therapy for COVID-19. But their side effects on liver and kidney and patients’ increasingly serious drug resistance rate cannot be ignored.

However, most of the treatments weaken or become ineffective as the virus strains change (Greaney et al., 2021; Cao et al., 2022b). The antiviral efficacy of lentinan with other COVID-19 antiviral drugs was not compared in this study, but the use of lentinan nasal drop in treatment has proven its safety and achieved good clinical antiviral efficacy, which is in line with the molecular mechanism and theoretical basis of enhancing mucosal immunity. At present, we are further studying the molecular mechanism of lentinan in the regulation of immunity and its anti-virus effect, including the investigation of other respiratory viruses.

Our study has limitations. In the first phase of the study, certain patients tested positive for nucleic acid 1–2 days before enrollment. Thus, a certain degree of bias possibly existed in the calculation of the virus shedding time. Next, in the second phase of the study, given the change in China’s domestic epidemic prevention policy, nucleic acid test was canceled, and nonsevere patients were not hospitalized and isolated. Therefore, we assessed patient results only by antigen test. Third, the 70 patients studied came from two schools in Shanghai. Most of them were young males, and thus, gender and age biases were possible. Finally, this study was a single-center research with a limited sample size. To further confirm the effectiveness of lentinan nasal drops in the treatment of COVID-19, we need to expand the sample size and conduct multicenter studies. In addition, when the dose was increased to 100 μg, the antiviral effect of lentinan drops was relatively significant, but the clinical symptoms showed no considerable improvement. The possible reasons from our analysis are as follows: 1. All the patients included in this study were young patients with mild symptoms and good tolerance. 2. The clinical symptom registries was filled out by patients themselves, and the answers were mostly based on their own subjective feelings rather than objective quantitative evaluation. Thus, bias was possible.

In summary, our study provides a new, economical, convenient, safe, and effective intervention method for the treatment and prevention of COVID-19. The proposed method has a strong transformation potential and can provide data and evidence support for the subsequent preparation of mucosal vaccines against respiratory infectious diseases such as coronavirus.

## Data Availability

The raw data supporting the conclusion of this article will be made available by the authors, without undue reservation.
