# Supplementary material for: Safety and efficacy of lentinan nasal drops in patients infected with the variant of COVID-19: a randomized, placebo-controlled trial
Source: Front Pharmacol. 2023 Dec 1;14:1292479. doi: 10.3389/fphar.2023.1292479 (PMC10722177; doi:10.3389/fphar.2023.1292479)
Supplement: Supplementary file 2 [file DataSheet1.docx]

Supplementary Material

**Safety and efficacy of lentinan nasal drops in patients infected with the variant of COVID-19**

Wenhan Fan^1†^, Benming You^2†^, Xinyu Wang^1†^, Xu zheng^3†^, Aijing Xu^1^, Yangang Liu^3^，Haoran Peng^3^，Wei Yin^1^，Mingxiao Xu^1^，Xu Dong^1^ Yayun Liu^1^，Ping Zhao^3*^, Xuesong Liang^1*^

^1^ Department of Infection Diseases，First Affiliated Hospital of Navy Military Medical University，Shanghai China

^2^ Department of Department of Pharmacy，First Affiliated Hospital of Navy Military Medical University，Shanghai China

^3^ Department of Microbiology, PLA Key Laboratory of Biodetection and Biodefense，Shanghai Key Laboratory of Medical Biodefense， Navy Military Medical University，Shanghai China

† These authors contributed equally to this work

*** Correspondence:**Xuesong Liang
liangxuesong2000@163.com

Ping Zhao

pnzhao@163.com

# Supplementary Data

**1.1 Main experimental methods、reagents and instruments**

**SARS-CoV-2 viral load** in nasopharyngeal swabs on alternate days since hospital admission was quantified by a commercially available RT-PCR test [Novel Coronavirus (2019-nCoV) Real Time RT-PCR Kit, cat. no. RR-0479–02, Liferiver Bio-Tech(China) Corp. Shanghai,China].

**Coronavirus (2019-nCoV) antigen** was tested by colloidal gold method [Novel Coronavirus (2019-nCoV) Antigen Rapid Detection kit, Bioscience(Tianjin) Diagnostic Technology Co. Ltd. Tianjin,China]

PCR-positive throat swab samples from the patients were used to isolate SARS-CoV-2 virus via Vero-E6 cell. The virus isolation was sequenced and submitted to GenBank (ON965380; ON965371; ON965362; ON965361).

1.2 Partial methods of animal experiments

Antibody detection in mice was performed according to ELISA techniques.(receptor binding domain,RBD) protein was on a high adsorption enzyme labeled microplate, 0.1 micrograms of protein per well plate,Overnight in a 4 ° C refrigerator.Removal the protein solution next day and wash the pore plate once with phosphate buffer solution (PBS, pH 7.0).PBS containing 3% bovine serum albumin (3% BSA PBS) was used seal the pore plate with room temperature for two hours.Subsequently, remove the sealing liquid and wash the hole 3 with PBS.Add continuously double diluted hamster serum to each well, with a dilution of 3% BSA-PBS and a volume of 100 μ L/well, and place it overnight in a refrigerator at 4 ° C; Remove serum diluent the next day.Wash the well plate 5 times with PBS containing 0.05% Tween 20 (0.05% Tween 20 PBS), and then add 1000 times diluted HRP labeled anti mouse IgG. The diluent is 3% BSA-PBS, with a volume of 100 μ l/well and leave at room temperature for 40 minutes.Absorb HRP antibody diluent and wash the plate well 5 times with 0.05% Tween 20-PBS;Add TMB chromogenic solution, with 100 microliters per plate well, for 10 minutes, and then add stop solution. Measure the light absorption values at 450 nm and 630 nm using an enzyme-linked immunosorbent assay (ELISA).RBD is the main target of neutralizing antibody against COVID-19, and the level of RBD antibody in serum represents the ability to fight against the virus.

# Supplementary Figures


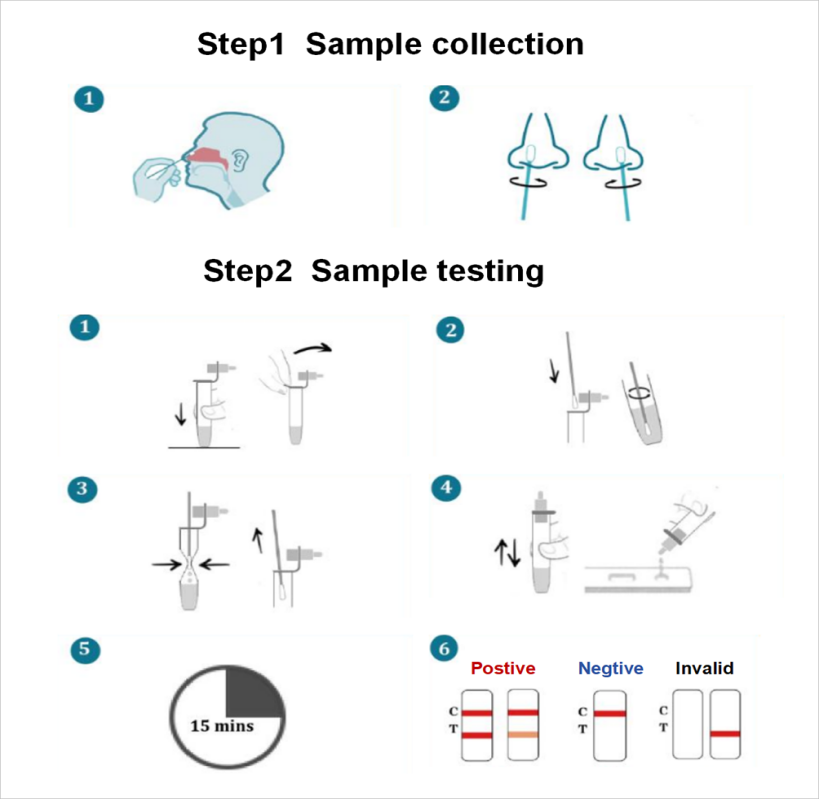


**Supplementary Figure 1.** Instruction of novel coronavirus (2019 nCoV) Antigen Detection Kit


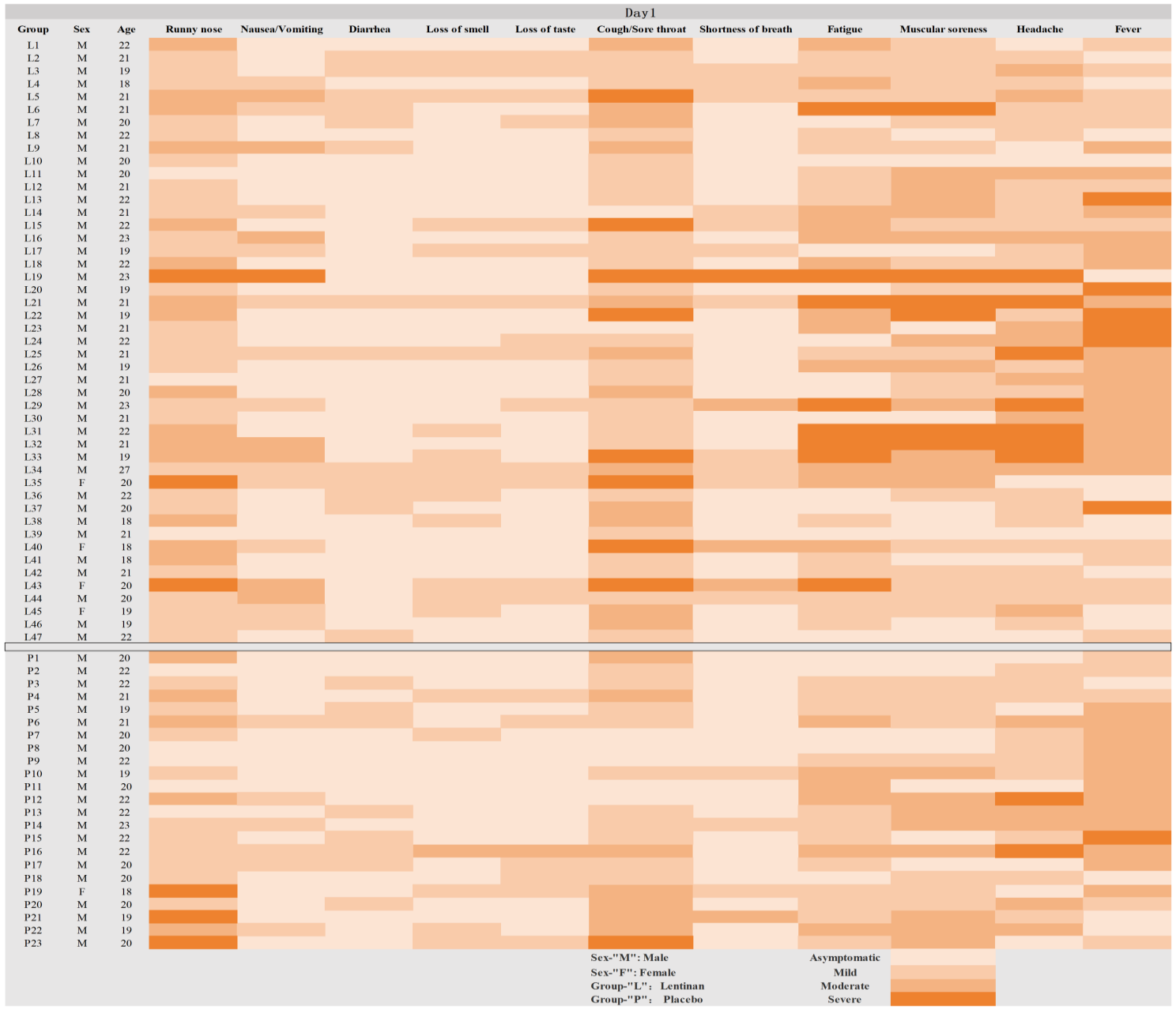


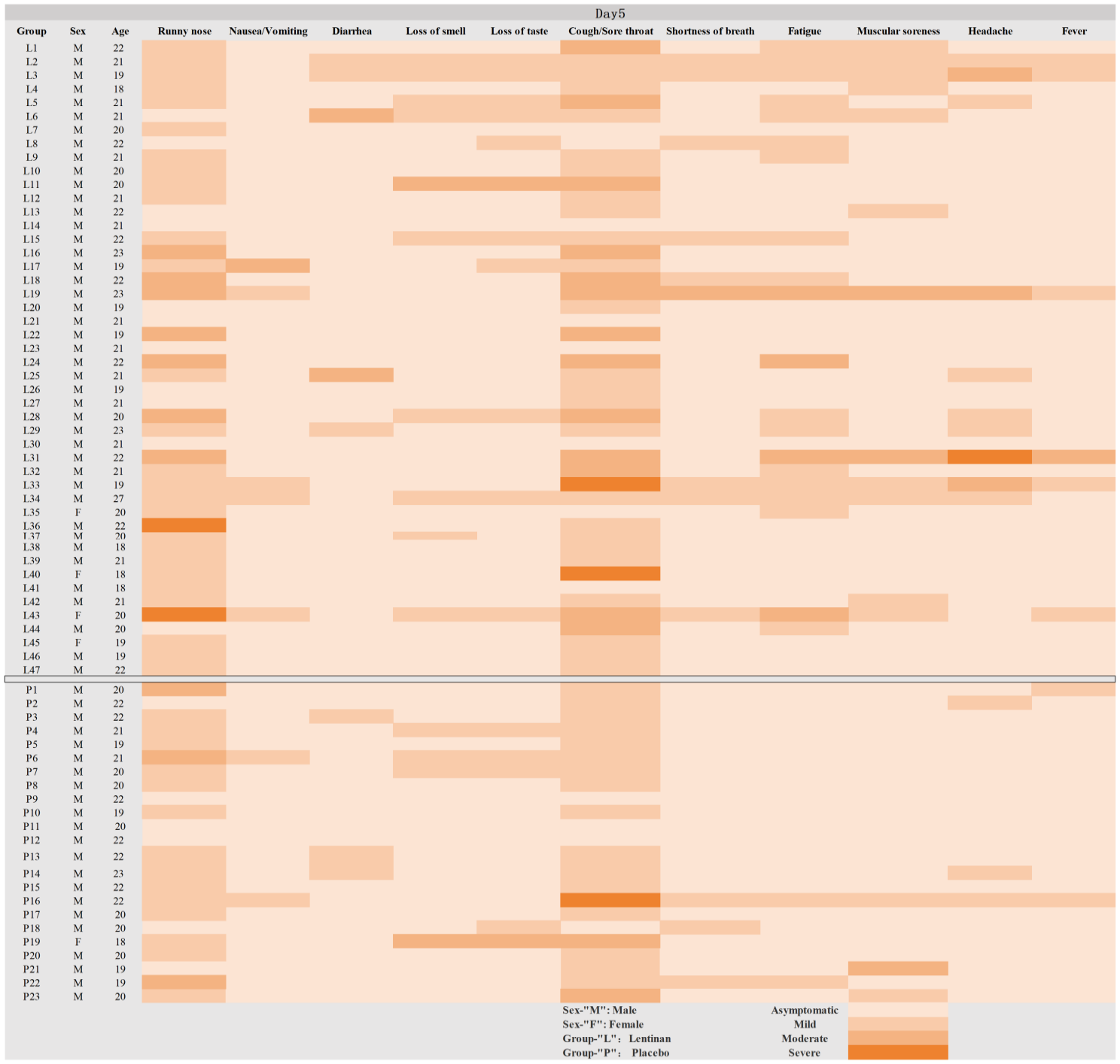


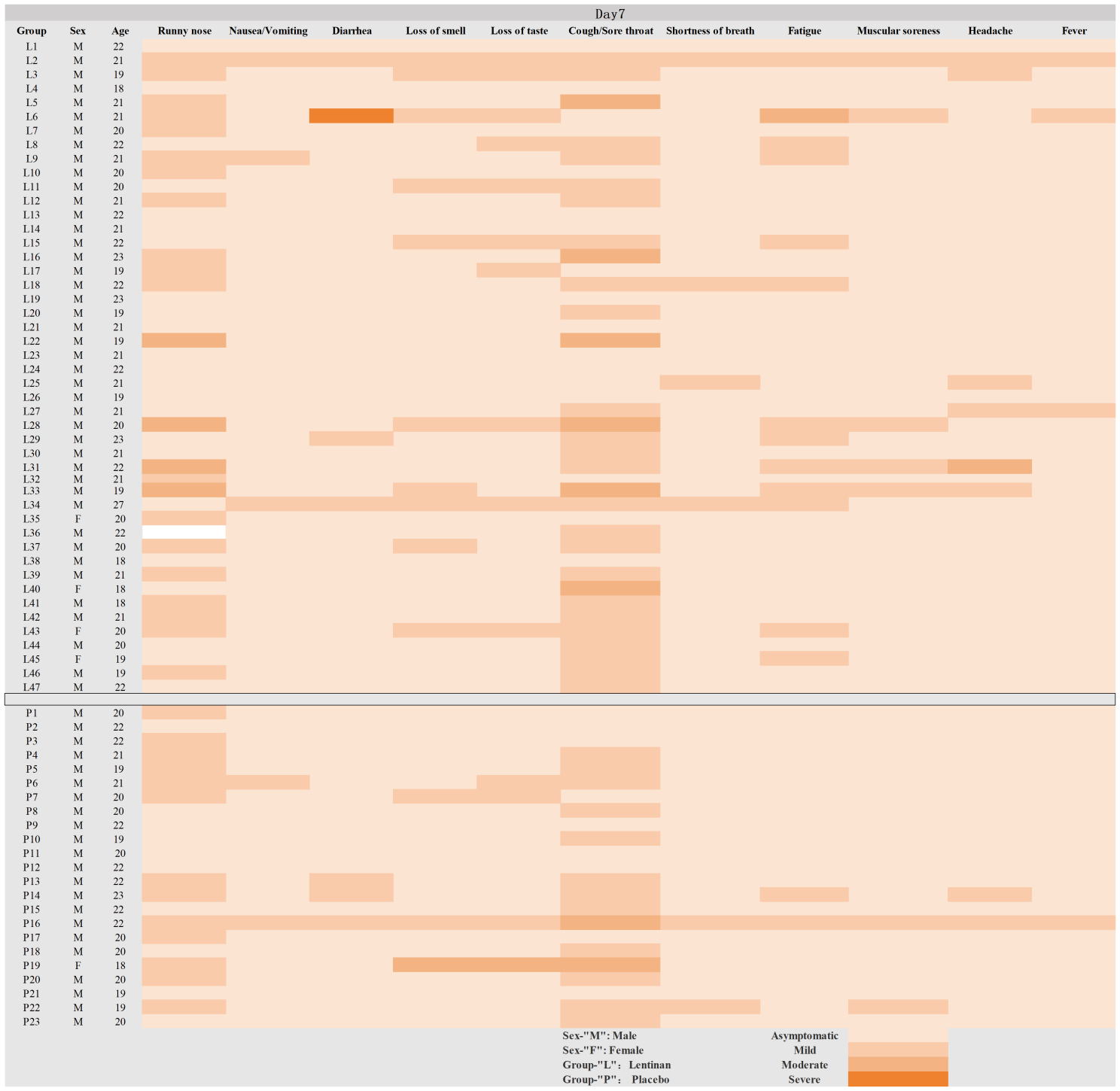


**Supplementary Figure 2.** Common clinical symptoms of the lentinan group and the placebo group at the different time points
